# Integrative ATAC-seq and RNA-seq Analysis of the Longissimus Dorsi Muscle of Gannan Yak and Jeryak

**DOI:** 10.3390/ijms25116029

**Published:** 2024-05-30

**Authors:** Zhidong Zhao, Dashan Guo, Yali Wei, Jingsheng Li, Xue Jia, Yanmei Niu, Zhanxin Liu, Yanbin Bai, Zongchang Chen, Bingang Shi, Xiaolan Zhang, Jiang Hu, Jiqing Wang, Xiu Liu, Shaobin Li

**Affiliations:** Gansu Key Laboratory of Herbivorous Animal Biotechnology, Faculty of Animal Science and Technology, Gansu Agricultural University, Lanzhou 730000, China; zhaozd@gsau.edu.cn (Z.Z.); guods@st.gsau.edu.cn (D.G.); weiyl@st.gsau.edu.cn (Y.W.); lijs@st.gsau.edu.cn (J.L.); jiax@st.gsau.edu.cn (X.J.); niuym@st.gsau.edu.cn (Y.N.); liuzx@st.gsau.edu.cn (Z.L.); baiyb@st.gsau.edu.cn (Y.B.); chenzongc@st.gsau.edu.cn (Z.C.); shibg@gsau.edu.cn (B.S.); zhangxl@gsau.edu.cn (X.Z.); wangjq@gsau.edu.cn (J.W.); liux@gsau.edu.cn (X.L.); lisb@gsau.edu.cn (S.L.)

**Keywords:** Jeryaks, ATAC-seq, RNA-seq, longissimus muscle, heterosis

## Abstract

Jeryak is the F1 generation of the cross between Gannan yak and Jersey cattle, which has the advantages of fast growth and high adaptability. The growth and development of skeletal muscle is closely linked to meat production and the quality of meat. However, the molecular regulatory mechanisms of muscle growth differences between Gannan yak and Jeryak analyzed from the perspective of chromatin opening have not been reported. In this study, ATAC-seq was used to analyze the difference of chromatin openness in longissimus muscle of Gannan yak and Jeryak. It was found that chromatin accessibility was more enriched in Jeryak compared to Gannan yak, especially in the range of the transcription start site (TSS) ± 2 kb. GO and KEGG enrichment analysis indicate that differential peak-associated genes are involved in the negative regulation of muscle adaptation and the Hippo signaling pathway. Integration analysis of ATAC-seq and RNA-seq revealed overlapping genes were significantly enriched during skeletal muscle cell differentiation and muscle organ morphogenesis. At the same time, we screened *FOXO1*, *ZBED6*, *CRY2* and *CFL2* for possible involvement in skeletal muscle development, constructed a genes and transcription factors network map, and found that some transcription factors (TFs), including YY1, KLF4, KLF5 and Bach1, were involved in skeletal muscle development. Overall, we have gained a comprehensive understanding of the key factors that impact skeletal muscle development in various breeds of cattle, providing new insights for future analysis of the molecular regulatory mechanisms involved in muscle growth and development.

## 1. Introduction

Jeryak represents the F1 generation resulting from the crossbreeding of Gannan yak and Jersey cattle, exhibiting distinct hybrid advantages including enhanced adaptability, reduced mortality rates, as well as accelerated growth and development [1]. The weight of Jeryaks in each period is reported to exhibit significant improvement compared to Gannan yaks. Specifically, the birth weight of Jeryaks is approximately 45–55% higher than that of Gannan yaks during the same period, while their weight at 12 months surpasses that of Gannan yaks by about 70–80% [2]. At the same time, cattle–yak meat is high in protein and lower in fat compared to yak meat, making it a suitable choice for a popular healthy and high-quality diet [3,4]. Therefore, it is of great significance to explore the regulatory mechanism of muscle growth and development in Jeryaks for genetic breeding improvement.

The growth and development of skeletal muscle is intricately linked to the yield and quality of meat [5]. The development of skeletal muscle in animals is a multifaceted process encompassing embryonic muscle fibers formation, postnatal hypertrophy, and adult regeneration [6]. Research has demonstrated that the growth and development of skeletal muscle is a physiological process regulated by various transcription factors and signaling pathways. The focus is particularly on the myogenic regulatory factor (MRF) family [7] and the myocyte enhancement factor-2 (MEF2) family [8], which play a crucial role in regulating skeletal muscle development. The presence of MEF2 and various MRFs can enhance muscle production [9]. Simultaneously, the Notch signaling pathway [10], Wnt signaling pathway [11] and mTOR signaling pathway [12] play a pivotal role in muscle proliferation and differentiation. Furthermore, the regulation of epigenetic mechanisms also plays a crucial role in the complex process of muscle development [13,14]. For example, m6 A modification positively regulates the expression levels of genes related to skeletal muscle growth and development at different developmental stages in Jeryaks, whereas it is negatively correlated with the expression levels of lncRNAs [15]. However, studies on skeletal muscle development in Gannan yaks and Jeryaks have mainly focused on phenotypic and transcriptional aspects, with limited analysis from an epigenetic regulatory perspective.

Eukaryotic genomic DNA is usually in the form of chromatin [16]. When chromatin regions are open, transcription factors (TFs) and other transcriptional regulators are permitted to access and activate gene expression. Conversely, when chromatin regions adopt a closed configuration, TFs binding to the transcriptionally active gene regions are hindered, resulting in gene silencing [17]. Chromatin accessibility refers to the extent of chromatin DNA molecules binding to regulatory elements, such as transcription factors (TFs), which can directly impact gene expression and transcriptional regulation [16,18,19]. The assay of transposase-accessible chromatin sequencing (ATAC-seq) is a novel technique for the detection of epigenomic profiles [20,21]. This technique detects chromatin accessibility by using Tn5 transposase and cuts exposed DNA regions for sequencing [22]. In recent years, it has gained significant traction in the exploration of cis-regulatory elements and the prediction of transcription factor binding sites [22]. Recently, ATAC-seq and RNA-seq techniques have been employed by researchers to investigate the skeletal muscle of various species including pigs and cows [23,24]. For instance, in Miao et al. [25], ATAC-seq and RNA-seq techniques were used to analyze and study the difference in skeletal muscle growth and development between Luchuan pigs and Duroc pigs. They predicted TFAP4, MAX, NHLH1, FRX5 and TGIF1 as potential factors influencing muscle growth and development. Additionally, they identified several candidate genes including *ASNS*, *CARNS1*, *G0S2*, *PPP1 R14 C* and *SH3 BP5*, through integration with RNA-seq data to potentially regulate muscle development. However, the relationship between chromatin accessibility and gene transcription regulation in the longissimus dorsi muscle of Gannan yak and Jeryak remains unexplored.

Therefore, in this study, we utilized ATAC-seq [26] to quantify transposase-accessible chromatin in order to investigate differences in chromatin open regions within the longissimus dorsi muscle tissue of various cattle breeds. The correlation between alterations in open chromatin regions and gene expression levels was further examined through the integration of RNA-seq data. These results will broaden our understanding of epigenetics during skeletal muscle development and provide a theoretical basis for further exploration of the underlying mechanisms of muscle development in both breeds of cattle.

## 2. Results

### 2.1. ATAC-seq Quality Control of the Gannan Yak and Jeryak Muscle Tissues

**Figure 1:**
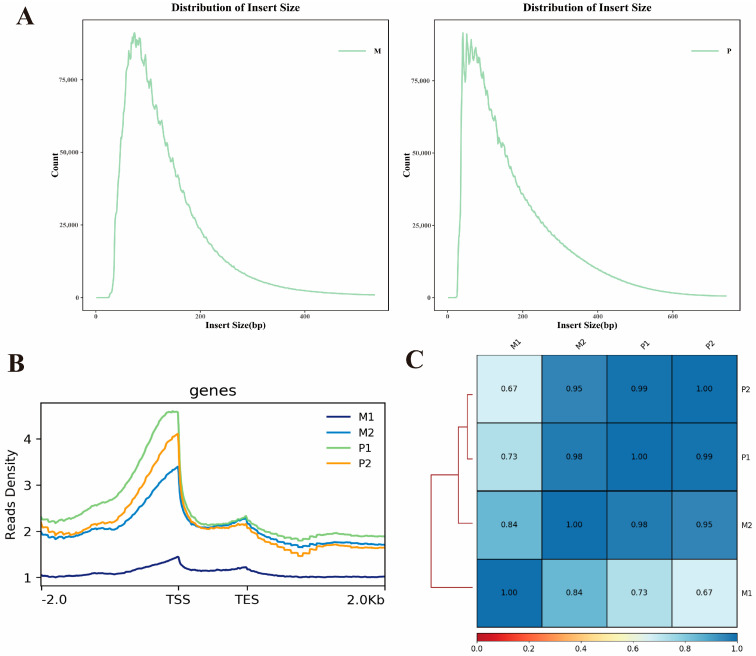
Quality control of ATAC-seq. (**A**) Distribution of insert sizes in groups M and P. (**B**) Enrichment of ATAC-seq signals is observed around the transcription start site (TSS). The *x*-axis represents the normalized length of gene or peaks, while the *y*-axis represents the degree of read enrichment. A higher value indicates a greater level of enrichment. TSS refers to transcription start site, and TES refers to transcription end site. The value −2.0 corresponds to 2 kb upstream of the TSS, and 2.0 corresponds to 2 kb downstream of the TES. (**C**) The heat map illustrates the results of Pearson correlation analysis.

In order to reveal the regulatory mechanisms behind the growth differences between the two breeds, we performed ATAC-seq studies on the longissimus dorsi muscle of Gannan yaks (M1 and M2) and Jeryaks (P1 and P2) to explore genomic chromatin accessibility. Totals of 91,332,714, 78,231,904, 116,244,208 and 99,271,020 original readings were obtained in M1, M2, P1 and P2, respectively. After filtration, 90,778,160, 77,589,728, 115,487,052 and 98,734,712 clean reads were obtained (Table 1). Meanwhile, 85.72% and 84.56% of clean reads in group M and group P were mapped to the yak reference genome (LU_Bosgru_v3.0), respectively (Table 2). We evaluated the quality of all sample libraries based on the length of the inserted fragment and the peak signal distribution. The analysis found that all libraries produced the expected effect, including abundant single nucleosome fragments and free nucleosome fragments (Figure 1A). This result is similar to previous studies [27]. Most of the identified chromatin accessibility regions were enriched at 2 kb of the transcription initiation site (Figure 1B), suggesting that the open chromatin region may play a role in transcriptional regulation. To further assess the quality of the ATAC-seq data, we conducted paired Pearson correlation analysis on the ATAC-seq samples, based on the read signal of the ATAC-seq peaks in all samples. The results revealed similarities within the same varieties and differences between different varieties (Figure 1C). All results indicate that the sequencing data are of high quality and can be further used for subsequent analysis.

### 2.2. Accessibility of Chromatin in the Longissimus Dorsi Muscle of Gannan Yaks and Jeryaks

**Figure 2:**
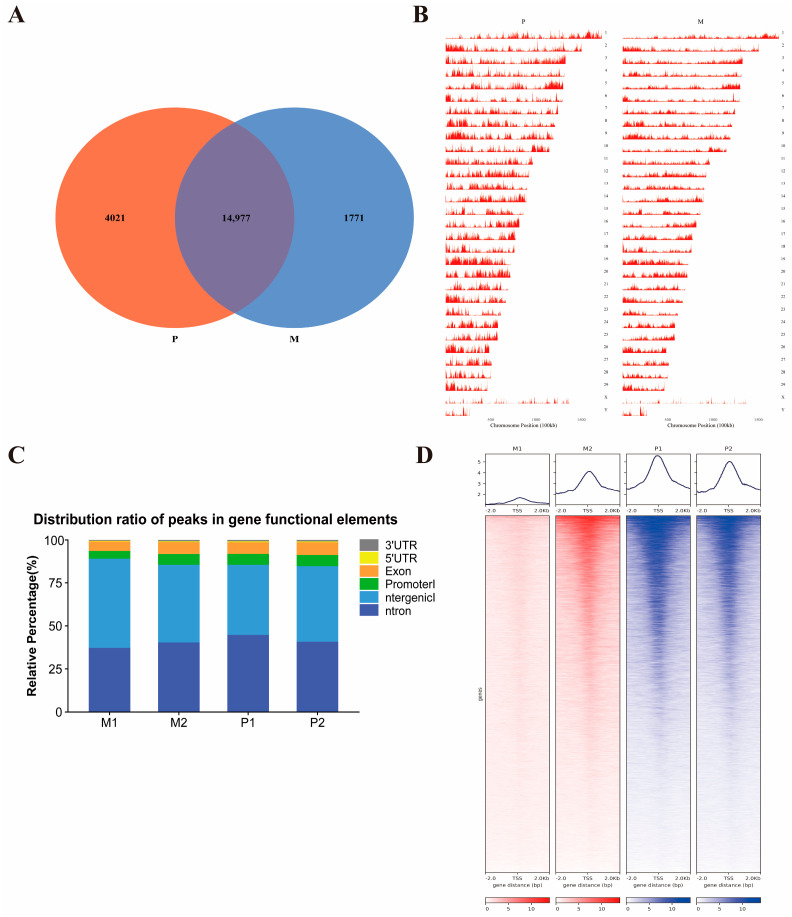
Identification and analysis of peaks. (**A**) Venn diagram illustrates peak overlap between M and P groups. (**B**) Chromosomal distribution of all peaks. (**C**) Distribution ratio of peaks in gene functional elements. Genomic functional regions comprise promoter, intergenic, exon, intron, 5’UTR and 3’UTR. (**D**) ATAC-seq signals were found to be enriched around the TSS in groups M and P. The above figure shows the enrichment map surrounding the TSS. The heat map illustrates the enrichment near the TSS, with ±2.0 representing regions upstream and downstream of the TSS.

We identified 1771 peaks specific to Gannan yaks, 4021 peaks specific to Jeryaks, and 14,977 common peaks (Figure 2A, Table S1). The distribution of peaks on the chromosomes of the Jeryak and Gannan yak genomes are shown in Figure 2B. It can be seen that most areas on all chromosomes, including the X and Y chromosomes, were covered by peaks. We annotated all the peaks using annotation files that categorize the genome-wide functional regions into introns, intergenic regions, promoters, exons, 5’UTR and 3’UTR (Figure 2C, Table S2). We found that most of the peaks were mapped to promoter, intron, exon and intergenic regions, and the peak of promoter regions in Gannan yak accounted for 3.426% of the total area, while the peak of promoter regions in Jeryak accounted for 5.565% of the total area. The heat map shows that the ATAC-seq signal intensity of Jeryak is much higher than that of Gannan yak, indicating that these strong signal peaks may be an important factor regulating muscle development (Figure 2D).

### 2.3. GO and KEGG Analysis of Genes Corresponding to Differential Peaks and Motif Analysis

**Figure 3:**
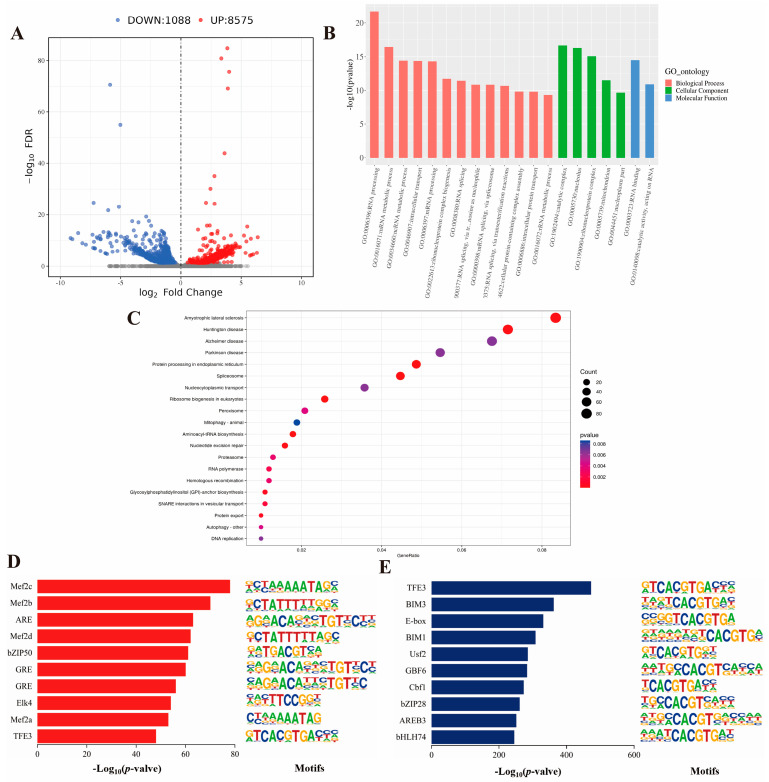
Functional enrichment analysis and motif analysis of differential peak-associated genes. (**A**) Volcano plot of differential peaks (FDR < 0.05, |Fold| > 0). The red dot represents the up-regulated difference peak and the blue dot represents the down-regulated difference peak. (**B**) GO enrichment analysis of differential peak-associated genes (*p* < 0.05). Red represents biological processes, green represents cellular components, and blue represents molecular functions. (**C**) KEGG enrichment analysis of differential peak-associated genes (*p* < 0.05). The dots range in color from 0 to 0.008. (**D**,**E**) The top 10 transcription factor binding motifs were enriched in significantly higher and significantly lower peak areas based on the *p*-values between the M and *p* groups, respectively.

To identify open chromatin sites associated with skeletal muscle growth and development, we conducted a comparison of peak differences between groups M and P using ATAC-seq. It was found that there were 8575 difference up-regulated peaks and 1088 difference down-regulated peaks in P compared to group M (Figure 3A). We obtained a total of 2663 genes by annotating the differential peaks, which corresponded to 2584 genes for the differential up-regulated peaks and 79 genes for the differential down-regulated peaks (Table S3). To investigate the potential functions of these genes, we analyzed these genes for GO and KEGG pathway enrichment. GO analysis showed that differentially expressed genes (DEGs) were enriched in negative regulation of muscle adaptation, regulation of organelle organization, metabolic processes and post-transcriptional regulation of gene expression (Figure 3B, Table S4). In addition, 40 KEGG pathways were significantly enriched (*p* < 0.05). Many of these pathways are involved in muscle development, such as the Hippo signaling pathway (Figure 3C, Table S5). The growth performance of Jeryak was reported to be superior to that of the Gannan yak [1]. Therefore, we used Homer software to analyze motifs on different peaks of the two species. The top 10 transcription factor binding motifs that showed a significant increase in peaks were found to be Mef2 family members, ARE, bZIP50, GRE, EIK4 and TFE3 (Figure 3D), whereas the top 10 enriched motifs with decreasing peaks contained TFE3, BIM3, E-box, Usf2, GBF6, Cbf1, bZIP28, AREB3 and bHLH74 (Figure 3E).

### 2.4. Integration Analysis of ATAC-seq and RNA-seq

**Figure 4:**
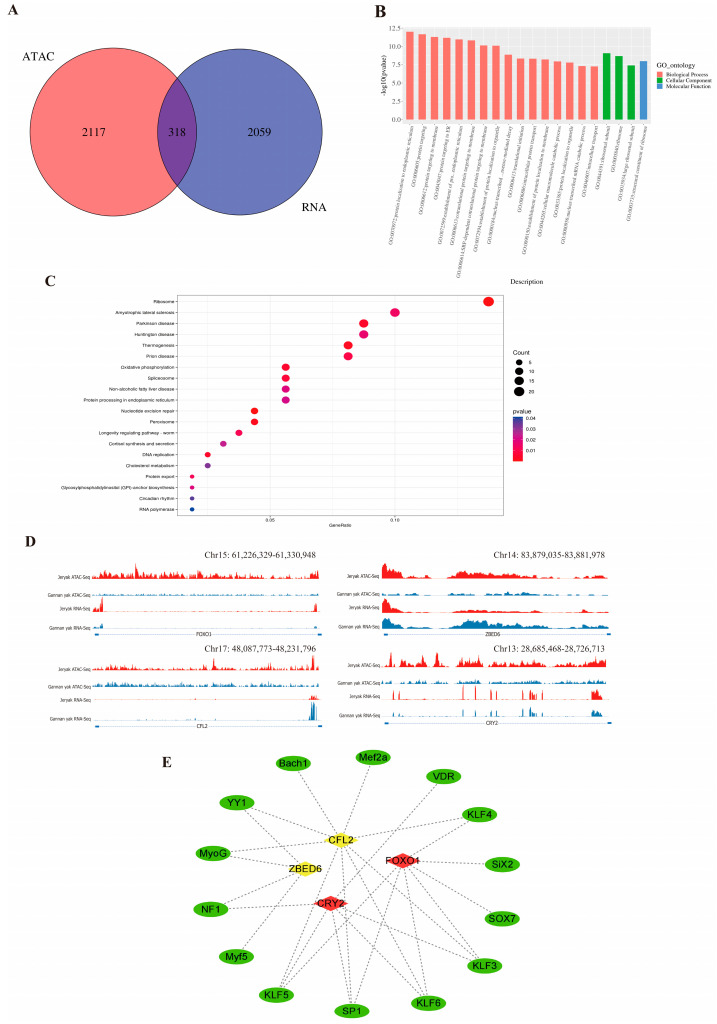
Results of ATAC-seq and RNA-seq combined analysis. (**A**) Venn map of differentially expressed genes identified by ATAC-seq and RNA-seq. (**B**) GO enrichment analysis of overlapping differential genes. Red represents biological processes, green represents cellular components, and blue represents molecular functions. (**C**) KEGG enrichment analysis of overlapping differential genes. The dots range in color from 0 to 0.04. (**D**) Visual display of ATAC-seq and RNA-seq signals in *FOXO1*, *CFL2*, *CRY2* and *ZBED6* genes. (**E**) Map of the interaction network between genes and transcription factors (TFs). The green oval represents TFs. Diamonds represent genes, red represents up-regulated genes, while yellow represents down-regulated genes.

To investigate the relationship between changes in open chromatin regions and gene expression levels, we conducted a combined analysis of ATAC-seq and RNA-seq data. A total of 318 differentially expressed genes (DEGs) were identified (Figure 4A, Table S6), and functional enrichment analysis showed that these DEGs were significantly enriched in processes such as skeletal muscle cell differentiation and muscle organ morphogenesis (Figure 4B,C, Table S7). To visualize how chromatin openness regulates gene expression, we used IGV to analyze ATAC-seq and RNA-seq signaling of genes related to muscle growth and development (*FOXO1*, *CFL2*, *CRY2* and *ZBED6*). The results revealed that the chromatin accessibility and transcript levels of *FOXO1* and *CRY2* were higher in Jeryaks than in Gannan yaks. In contrast, the chromatin accessibility of *CFL2* and *ZBED6* in Jeryaks was higher than that in Gannan yaks, but the transcript levels were lower (Figure 4D). To identify key genes, we predicted transcription factor binding sites within ±2 kb of the transcription start site using FIMO software. Based on this, we constructed a network map of the interactions between the 4 key genes and 14 transcription factors through a literature search (Figure 4E). The genes were found to closely interact with transcription factors (TFs) and are likely to play significant roles in regulating the growth and development of skeletal muscle.

### 2.5. Integration Validation of the Results by qRT-PCR

**Figure 5:**
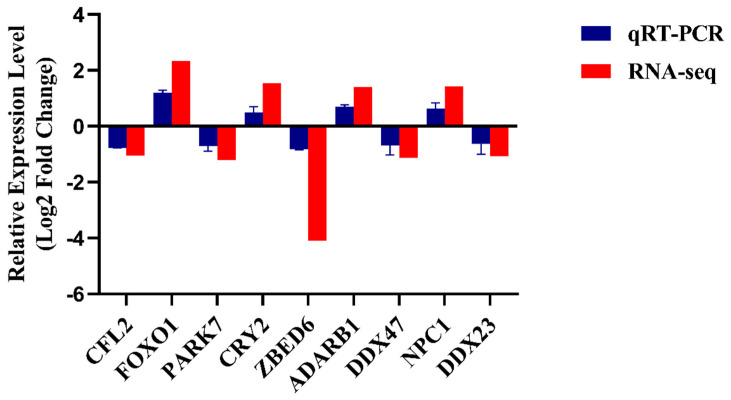
Validation of sequencing results was performed using qRT-PCR. The histogram depicts the expression levels of 9 genes by RNA-seq and qRT-PCR. The *x*-axis represents the 9 DEGs, while the *y*-axis represents the gene expression level.

To verify the accuracy of the RNA sequencing data, we randomly selected nine overlapping genes (*FOXO1*, *CFL2*, *CRY2*, *DDX23*, *ADARB1*, *DDX47*, *ZBED6*, *PARK7* and *NPC1*) from the joint analysis for qRT-PCR analysis. The results indicate that the gene expression patterns of Gannan yak and Jeryak were in line with the sequencing findings (Figure 5). This finding further reinforces the credibility of our sequencing data.

## 3. Discussion

The meat yield, influenced by the growth and development of skeletal muscle, is a crucial determinant in assessing the economic value of livestock [28]. The growth performance and other aspects of Jeryak, as a hybrid F1 generation, were reported to be superior to those of Gannan yak [29]. Therefore, elucidating the molecular mechanisms underlying the regulation of skeletal muscle growth and development is a crucial prerequisite for expediting genetic breeding and enhancement of Gannan yak, thereby optimizing economic benefits for herders. In recent years, research efforts have primarily focused on the transcriptional regulation and m6 A methylation of yak and cattle–yak [15,30], but few studies have been conducted from the perspective of chromatin opening. To this end, we employed the ATAC-seq analysis method to elucidate the disparities in muscle growth and development between Gannan yak and Jeryak by investigating chromatin accessibility. Additionally, we integrated RNA-Seq data to identify pivotal factors potentially influencing muscle development.

In this study, we observed a pronounced enrichment of ATAC signal peaks near the transcription start site (TSS) in groups P and M, indicating their potential significance as key regulatory nodes for transcription factor binding and transcriptional regulation. This is consistent with previous studies of chromatin accessibility during early embryogenesis in five species [31]. In addition, the peaks observed in the M and P groups exhibited a higher proportion within intergenic regions and introns, followed by exons, promoters, 3’UTR and 5’UTR. The findings align with the investigation conducted by Cao et al., which explored chromatin accessibility in testicular tissue of yak and cattle–yak [32]. We hypothesized that this might be due to the high intergenic and intron ratios in the yak genome [33].

We conducted a comprehensive analysis of the dynamic changes in chromatin accessibility across different varieties. ATAC-seq analysis revealed 1088 down-regulated differential peaks and 8575 up-regulated differential peaks in group P compared to group M. Subsequently, functional enrichment analysis of the differentially peaked annotated genes revealed their predominant enrichment in negative regulation of muscle adaptation, regulation of organelle organization, metabolic processes, post-transcriptional gene expression regulation, and signaling pathways related to muscle development, such as the Hippo signaling pathway. Previous studies have identified the Hippo signaling pathway as a pivotal regulator of skeletal muscle development and regeneration [34]. A previous study showed that the Hippo pathway affects muscle growth by regulating the expression of sarcoma genes that control muscle fiber growth in skeletal muscle [35]. Recent studies have demonstrated that Yap and Taz, as effectors of the Hippo signaling pathway, play a crucial role in regulating the proliferation and differentiation of myoblasts [36]. Notably, several transcription factors implicated in muscle growth were identified among the predicted transcription factor binding motifs that exhibited either an increase or decrease in peaks, such as TFE3, MEF2 D, MEF2 A and MEF2 C. Previous studies have demonstrated that adenovirus-mediated TFE3 overexpression impedes myoblast differentiation and reduces the expression of muscle regulatory factors (MRFs) such as MYOD and MYOG, whereas TFE3 knockout has the opposite effect, indicating that TFE3 modulates myoblast differentiation by regulating the expression of myogenic marker genes [37]. It is well known that members of the MEF2 family of transcription factors have an essential role in skeletal muscle growth, myocyte proliferation and differentiation [8,38,39,40,41]. The transcription factor MEF2 A has been reported to regulate the development of skeletal muscle by binding to the promoter region of the bovine *LATS2* gene [42]. Early studies have also demonstrated that *MEF2 C* knockout in mice leads to rapid muscle fiber lesions, thereby disrupting skeletal muscle development, whereas specific knockouts of MEF2 A or MEF2 D do not exhibit any abnormalities in skeletal muscle development [43]. Furthermore, individual knockouts of MEF2 A, C, or D did not exhibit any discernible impact on the process of skeletal muscle regeneration; however, simultaneous knockout of MEF2 A, C, and D resulted in a compromised regenerative capacity of skeletal muscle [44].

We integrated ATAC-seq and RNA-seq data to further explore the potential correlation between chromatin accessibility and gene expression levels. A total of 318 DEGs were found to be co-expressed in ATAC-seq and RNA-seq. The functional enrichment analysis revealed that the differentially expressed genes (DEGs) were significantly enriched in processes related to skeletal muscle cell differentiation and muscle organ morphogenesis. We hypothesized that this might be one of the factors affecting the growth difference between the two varieties. Next, we focus on four genes: *FOXO1*, *CRY2*, *ZBED6* and *CFL2*. They have been shown to be involved in muscle growth and development. It has been reported that the knockdown of *FOXO1* significantly enhances the proliferation and differentiation of bovine myoblasts while inhibiting their apoptosis. In addition, *FOXO1* interacts with genes associated with myogenesis to regulate the process [45]. He et al. [46] found that the loss of *CRY2* led to a significant increase in PAX7 expression and enhanced the proliferation of satellite cells, thus promoting muscle generation. Meanwhile, Liu et al. [47] found that *Zbed6*+/− may promote muscle growth in mice by upregulating *Barx2*. Additionally, *CFL2*, serving as a crucial mediator in promoting bovine myogenic differentiation, facilitates the differentiation of bovine myoblasts through the regulation of muscle differentiation marker gene expression [48]. The signal visualization revealed a consistent trend in the signals of ATAC-seq and RNA-seq for *FOXO1* and *CRY2*, both exhibiting robust signals in Jeryak. We believe that there is a positive correlation between chromatin openness and gene expression levels. This is consistent with the findings of chromatin accessibility in bovine oocytes and early embryos [49]. It is noteworthy that *ZBED6* and *CFL2* have opposite signaling trends, which may be influenced by histone modifications or other epigenetic modifications. In addition, it is possible that there are certain inhibitory TFs that result in restricted transcription of genes. Based on these findings, we hypothesized that four genes, *FOXO1*, *CRY2*, *ZBED6* and *CFL2*, may be key regulators influencing muscle growth differences between the two breeds of cattle. On this basis, by predicting its transcription factors, it was found that YY1, KLF4, KLF5, Bach1 and other transcription factors were involved in skeletal muscle development. Notably, YY1 is a widely expressed transcription factor in mammalian tissues and has been implicated in the regulation of diverse developmental and differentiation processes [50]. Recent studies have shown that in mutant mice, knocking out the transcription factor YY1 blocks the differentiation of skeletal muscle satellite cells, resulting in impaired skeletal muscle regeneration [51]. KLF4 and KFL5, members of the Kruppel-like transcription factor family, primarily participate in regulating cellular proliferation and differentiation processes [52]. KLF4 suppresses myoblast proliferation by binding to specific motifs on the *P57* promoter region, whereas it regulates myoblast fusion through activation of Myomixer expression [53]. KLF5 may play a role in the Wnt/β-catenin signaling pathway in the regulation of skeletal muscle atrophy, as demonstrated in an in vitro assay using chicken satellite cells [54]. Furthermore, in C2 C12 cells, we observed that Bach1 facilitates myoblast differentiation by suppressing the expression of *Smad2* and *Smad3* [55]. Previous studies have demonstrated that transcription factors (TFs) exert their influence on gene expression by selectively binding to specific cis-regulatory elements within the target gene region, thereby modulating gene expression levels [56]. Therefore, we constructed a network of interactions between genes and TFs and observed that genes in the regulatory network can interact with multiple TFs. In future studies, we should further validate the potential regulatory mechanisms of TFs by key genes for muscle development and their effects on skeletal muscle growth and development.

## 4. Materials and Methods

### 4.1. Experimental Animals and Sample Collection

Two male Gannan yaks (M1 and M2) and two male Jeryaks (P1 and P2) were obtained from Hezuo City, Gannan Tibetan Autonomous Prefecture, Gansu Province, China. All animals were 4 years old. They had free access to food and water under the same conditions. After being approved by Animal Policy and Welfare Committee of Gansu Agricultural University (Ethic approval file No. GSAU-Eth-AST-2023-001). Before slaughter, they go through an overnight fasting period. Following slaughter, samples of longissimus dorsi muscle were collected and promptly frozen in liquid nitrogen for subsequent experiments.

### 4.2. ATAC-seq Library Preparation, Sequencing and Analysis

ATAC-seq was performed as described by Wu et al. [57]. In brief, a small amount of longissimus dorsi muscle tissue was collected and ground to a powder in a pre-cooled cracking buffer. Then, the nucleus was subsequently isolated by centrifugation at 4 °C and 2200× *g* for 15 min. The transposition was performed at 37 °C for 30 min with 2.5 μL Nextera Tn5 transposition enzyme (Illumina, CA, USA). After the translocation reaction, the DNA was purified using the Qiagen MinElute PCR Purification Kit (Tiangen Biotech, Beijing, China). The NEBNext High-Fidelity 2 × PCR Master Mix was expanded to 10–15 cycles [58]. The amplified libraries were purified by Qiagen MinElute PCR purification kit (Tiangen Biotech, Beijing, China). The libraries were quality-checked using Agilent 2100 to complete the library construction. Finally, sequencing was conducted using the Illumina NovaSeq 6000 platform located in Wuhan, China.

The raw data obtained from sequencing were stored in the FASTQ file format. Among them, the sequence and corresponding sequencing quality information were included. The fastq [59] (v 0.11.5, https://www.bioinformatics.babraham.ac.uk/projects/fastqc, accessed on 27 October 2023) software was used to filter adapters and remove joint sequences, contaminated sequences, low-quality bases and reads to obtain high quality clean reads. The clean reads obtained after data quality control were aligned to the yak reference genome (LU_Bosgru_v3.0), as well as the mitochondria and chloroplasts, using hisat2 [60] (v2.1.0, https://www.ccb.jhu.edu/people/infphilo, accessed on 27 October 2023) software. Subsequent analysis excluded reads originating from chloroplasts and mitochondria. 

The samtools [61] (v1.9, https://www.htslib.org, accessed on 27 October 2023) software and MACS [62] (v2.1.0, https://hbctraining.github.io/Intro-to-ChIPseq/lessons/05_peak_calling_macs.html, accessed on 27 October 2023) software were used to remove potential PCR duplicate samples and perform peak detection to obtain a genome-wide profile of open chromatin regions for each sample. The ChIPseeker R package (https://guangchuangyu.github.io/software/ChIPseeker, accessed on 27 October 2023) was used to map the distribution of reads over gene functional elements. The deeptools [63] (v2.5.4, https://deeptools.readthedocs.io/en/develop, accessed on 27 October 2023) software was utilized to quantify the read distribution upstream and downstream of the transcription start site (TSS) within a 2 kb range, followed by generating density plots and heat maps. The Diffbind [64] (v1.16.3, https://bioconductor.org/packages/release/bioc/html/DiffBind.html, accessed on 27 October 2023) software was employed to identify differential peaks, which were considered gene-associated if they resided within a 5000 bp proximity of the gene’s Transcription Start Site (TSS). Difference peaks were considered up-regulating if they had an FDR < 0.05 and Fold > 0, while down-regulating difference peaks were defined by an FDR < 0.05 and Fold < 0. Homer [65] (v3, http://homer.ucsd.edu/homer, accessed on 27 October 2023) software was employed for the prediction of motifs in differentially enriched peak sequences. For ATAC-seq, two biological replicates were used.

### 4.3. GO and KEGG Enrichment Analysis of Differential Peak-Associated Genes

We found the nearest gene within 5 kb of the midpoint of the peak of significant difference. GO and KEGG annotations were made using the R package ClusterProfiler4.0 [66], with significance thresholds set at *p* < 0.05 for GO terms and identification of KEGG pathways.

### 4.4. Integration Analysis of ATAC-seq and RNA-seq

Next, we integrated RNA-seq data (accession number: PRJNA1023693) for further analysis. The results of genome-wide visualization of ATAC-seq and RNA-seq intersections were presented using the IGV (v2.12.2, https://software.broadinstitute.org/software/igv/download, accessed on 27 October 2023) software. The prediction of transcription factor binding sites was performed using FIMO [67] (v 4.11.2, https://meme-suite.org/meme/doc/fimo.html, accessed on 27 October 2023) software.

### 4.5. Identification of Differentially Expressed Genes by qRT-PCR

To validate the accuracy of the sequencing results, we randomly selected 9 genes for qRT-PCR verification. The primers were designed using Primer 5.0, with *GAPDH* serving as the internal reference gene. The primer sequence information was presented in Table 3 with detailed elaboration. RNA extraction was initially conducted using TRIzol reagents (Invitrogen, Carlsbad, CA, USA), followed by cDNA synthesis performed with a reverse transcription kit (Vazyme, Nanjing, China). In brief, a 16 µL reaction system was prepared by incorporating a 4 × gDNA wiper Mix, Nuclease-free water, and diluted RNA into the PCR tube. The reaction was gently blown and mixed, followed by heating at 42 °C for 2 min. Subsequently, a 20 µL system was prepared by adding 5 × HiScript II qRT SuperMix II, followed by cDNA synthesis through incubation at 50 °C for 15 min and subsequent heating at 85 °C for 5 s. Subsequently, a fluorescence quantitative kit (Transgen, Beijing, China) was employed for conducting the fluorescence quantitative PCR assay. The cDNA, Forward Primer (10 µM), Reverse Primer (10 µM), 2 × PerfectStart^®^Green qPCR SuperMix, Passive Reference Dye (50×), and Nuclease-free water were combined in a new PCR tube to create a 20 µL reaction system. The real-time fluorescence quantitative PCR was performed for 40 to 45 cycles at 94 °C for 5 sec and 60 °C for 30 sec. We performed qRT-PCR on the ABI Prism 7500 real-time fluorescent quantitative PCR system (Applied Biosystems, Foster City, CA, USA). The 2^−ΔΔCT^ method was employed for data analysis, and all experiments were conducted with three biological replicates.

### 4.6. Statistical Analysis

The results of qRT-PCR analysis were expressed as mean ± SD, and *p* < 0.05 was the criterion for screening functional enrichment pathways. GraphPad Prism 9 software was utilized for data visualization. The co-expression network of genes and transcription factors (TFs) was constructed using Cytoscape (v 3.10.1, htpps://cytoscape.org, accessed on 27 October 2023).

## 5. Conclusions

In conclusion, our study has conducted a preliminary exploration of the chromatin accessibility in the longissimus dorsi muscle of Gannan yak and Jeryak. Through the combined analysis of ATAC-seq and RNA-seq, we screened four muscle growth-related genes (*FOXO1*, *CFL2*, *CRY2* and *ZBED6*), and the differential expression of these genes might be one of the reasons for the growth differences between Jeryaks and Gannan yaks. In addition to this, we constructed a network diagram of the interactions between muscle growth-related genes and transcription factors (TFs). These findings provide a foundation for further studies on the molecular regulatory mechanisms of skeletal muscle growth and development in Gannan yaks and Jeryaks.

## Figures and Tables

**Table 1:** ATAC-seq data statistics.

Sample	Raw Reads	Raw Bases	Clean Reads	Clean Bases	Clean Ratio	Q20	Q30	GC
M1	91,332,714	13,699,907,100	90,778,160	9,844,432,268	99.39%	98.65%	95.57%	49.01%
M2	78,231,904	11,734,785,600	77,589,728	9,045,505,914	99.18%	98.33%	94.78%	50.65%
P1	116,244,208	17,436,631,200	115,487,052	13,089,463,824	99.35%	98.40%	94.88%	50.53%
P2	99,271,020	14,890,653,000	98,734,712	10,698,377,169	99.46%	98.38%	94.76%	49.35%

**Table 2:** Reference gene matching results.

Sample	Total Reads	Mapped Reads	Map Rate	Uniq	Paired
M1	90,778,160	77,361,144	85.22%	62,555,109	60,227,766
M2	77,589,728	66,895,805	86.22%	56,009,360	53,197,036
P1	115,487,052	98,334,567	85.15%	83,789,052	79,604,294
P2	98,734,712	82,898,529	83.96%	69,904,662	66,443,372

**Table 3:** Primers of mRNA used for the qRT-PCR.

mRNA	Forward (5′ → 3′)	Reverse (5′ → 3′)
*FOXO1*	ACCCCACAAGGTTTCCGATG	AGTGTCCCCTCTCTTTCCAAC
*CFL2*	ATTCTGGGCTCCTGAAAGTGC	TCTCTCCAAGTGTGGAACGG
*ZBED6*	ACTGGACAAGGGCCAACAAA	AAGCTTCCACTGCTTCCAGC
*PARK7*	TAAGGTCACCGTTGCAGGTC	CCTCCTGGAAGAACCACCAC
*DDX23*	AACGGCATCGTTCAAGGGAT	TCCCTGTCTCGGTCCTTCTT
*DDX47*	TCTGCCCATTCTCAACGCAT	CAATGACAGCACACTGCACC
*ADARB1*	AGCTGAACGAGATCAAGCCC	CTCGAACACCTGTCCGTTGA
*NPC1*	TGCCACAGGATGGCTATGAC	GGACAGAAACTGCAGAGGCA
*CRY2*	GATTCCCCACTGAAGGGCTC	GCGGTAGTAGAAGAGACGGC
*GAPDH*	CCACGAGAAGTATAACAACACC	GTCATAAGTCCCTCCACGAT

## Data Availability

The RNA-seq data from this study are available in GenBank Sequence Read Archive (SRA) database with accession number PRJNA1104381 (accessed 10 June 2027) and PRJNA1023693 (accessed 10 June 2027).

## Supplementary Materials

The following supporting information can be downloaded at: https://www.mdpi.com/article/10.3390/ijms25116029/s1.
